# Bioinformatics analysis reveals shared molecular pathways for relationship between ulcerative colitis and primary sclerosing cholangitis

**DOI:** 10.1186/s44342-025-00045-4

**Published:** 2025-05-15

**Authors:** Pooya Jalali, Malihe Rezaee, Alireza Yaghoobi, Moein Piroozkhah, Mohammad Reza Zabihi, Shahram Aliyari, Zahra Salehi

**Affiliations:** 1 Basic and Molecular Epidemiology of Gastrointestinal Disorders Research Center, Research Institute for Gastroenterology and Liver Diseases, Shahid Beheshti University of Medical Sciences, Tehran, Iran; 2 Department of Pharmacology, School of Medicine, Shahid Beheshti University of Medical Sciences, Tehran, Iran; 3 Department of Immunology, School of Medicine, Tehran University of Medical Sciences, Tehran, Iran; 4 Division of Applied Bioinformatics, German Cancer Research Center DKFZ Heidelberg, Heidelberg, Germany; 5 Hematology, Oncology and Stem Cell Transplantation Research Center, Research Institute for Oncology, Hematology and Cell Therapy, Tehran University of Medical Sciences, Tehran, Iran

**Keywords:** Inflammatory bowel disease, Ulcerative colitis, Primary sclerosing cholangitis, Bioinformatics

## Abstract

**Background:**

Inflammatory bowel disease (IBD) is a group of chronic inflammatory disorders, including ulcerative colitis (UC) and Crohn’s disease, affecting the gastrointestinal tract and is associated with high morbidity and mortality. Accumulating evidence indicates that IBD not only impacts the gastrointestinal tract but also affects multiple extraintestinal organs, which may manifest prior to the diagnosis of IBD. Among these extraintestinal manifestations associated with IBD, primary sclerosing cholangitis (PSC) stands out as a prominent example. PSC is recognized as a progressive cholestatic disorder, characterized by the narrowing of bile ducts, eventual development of liver cirrhosis, end-stage liver disease, and the potential emergence of cholangiocarcinoma. This study aimed to identify the molecular contributors in UC-induced PSC by detecting the essential regulatory genes that are differentially expressed in both diseases.

**Materials and methods:**

The common single-nucleotide polymorphisms (SNPs) and differentially expressed genes (DEGs) were detected using DisGeNET and GEO databases, respectively. Then, the top module and hub genes within the protein–protein interaction network were identified. Furthermore, the co-expression network of the top module was constructed using the HIPPIE database. Additionally, the gene regulatory network was constructed based on miRNAs and circRNAs. Finally, we searched the DGIdb database for possible interacting drugs with UC-PSC top module genes.

**Results:**

A total of 132 SNPs and their associated genes were found to be shared between UC and PSC. Gene expression analysis identified 56 common DEGs between the two diseases. Following functional enrichment analysis, 207 significant biological processes (BP), 48 molecular functions (MF), and 8 KEGG pathways, with notable enrichment in mRNA-related processes such as mRNA splicing and RNA binding, were defined. Particularly, the PTPN2 gene was the only gene common between UC and PSC at both the SNP level and the expression level. Additionally, the top cluster of PPI network analysis was consisted of PABPC1, SNRPA1, NOP56, NHP2L1, and HNRNPA2B1 genes. Finally, ceRNA network involving 4 mRNAs, 94 miRNAs, and 200 selected circRNAs was constructed.

**Conclusion:**

The present study provides novel potential candidate genes that may be involved in the molecular association between ulcerative colitis and primary sclerosing cholangitis, resulting in the development of diagnostic tools and therapeutic targets to prevent the progression of PSC from UC.

**Supplementary Information:**

The online version contains supplementary material available at 10.1186/s44342-025-00045-4.

## Introduction

Inflammatory bowel disease (IBD) is a public health issue which is associated with high morbidity and mortality. It has been reported that the global prevalence and burden of IBD increased over recent decades; The prevalence rate of IBD elevates from 79.5 to 84.3 per 100,000 population during 1990–2017 period [1]. IBD is a chronic idiopathic disorder of the gastrointestinal (GI) tract, characterized by excess inflammatory responses in GI tract. Crohn’s disease (CD) and ulcerative colitis (UC) are two major categories of IBD [2]. Accumulating evidence indicated that IBD not only affects the GI tract but also it involves various extraintestinal organs which may present before IBD diagnosis [3]. Extraintestinal manifestations (EIMs) can occur in up to 50% IBD cases [4], which 24% of those may manifest before the onset of intestinal features [3].

Primary sclerosing cholangitis (PSC) is one of the most IBD-associated EIMs, which is known as a progressive cholestatic disease. Triggering the inflammation and fibrosis of intrahepatic and extrahepatic biliary tree contributes to the pathogenesis of PSC, which may eventually lead to strictures of bile ducts, liver cirrhosis, end-stage liver disease, and cholangiocarcinoma [5–7]. The prevalence of PSC is about 2–8% and 3.5% among patients with UC and CD, respectively. In contrast, the presence of IBD, most frequently UC, has been reported in approximately 70% of patients with PSC [7, 8]. A strong association between PSC and UC was found which may be resulted from interrelated underlying mechanisms. For instance, antibodies against cytoplasmic and nuclear antigens of neutrophils with a characteristic perinuclear staining pattern (p-ANCA) have been commonly found in both patients with UC and PSC [9]. High-throughput DNA sequencing technologies and gene chip experiments have enabled high potential investigation fields to screen and compare differentially expressed genes (DEGs) in pathologic and normal tissue cells [10]. Recently, growing body of large-scale genome-wide association studies (GWAS) have identified a strong role of genetic components in the pathogenesis of both PSC and IBD [11, 12]. Furthermore, several studies have found the genome-wide genetic relationship between PSC and IBD, particularly UC [13, 14]. However, the exact underlying mechanisms of the pathogenesis of UC-induced PSC have remained poorly understood.

Due to the strong evidence concerning UC and PSC association and limited data regarding the genomic relationship between them, we aimed to identify the various aspects of possible molecular correlations between these two conditions. In this way, we detected the common genes significantly differentiated and common SNPs in UC and PSC, using GEO and DisGeNET datasets, respectively. Then, functional enrichment analysis and protein–protein network construction were performed, and top module was determined. Finally, long noncoding RNAs (lncRNAs), competitive endogenous RNA (ceRNA) network, and co-expression network related to the genes of top module were assessed. Our study offers a comprehensive insight into the function of commonly expressed differentially expressed genes in inducing PSC following UC. Our analyses have the potential to pave the way for the development of novel pharmaceuticals and treatments that can more effectively target omics, as well as improve preventative care measures.

## Materials and methods

**Fig. 1:**
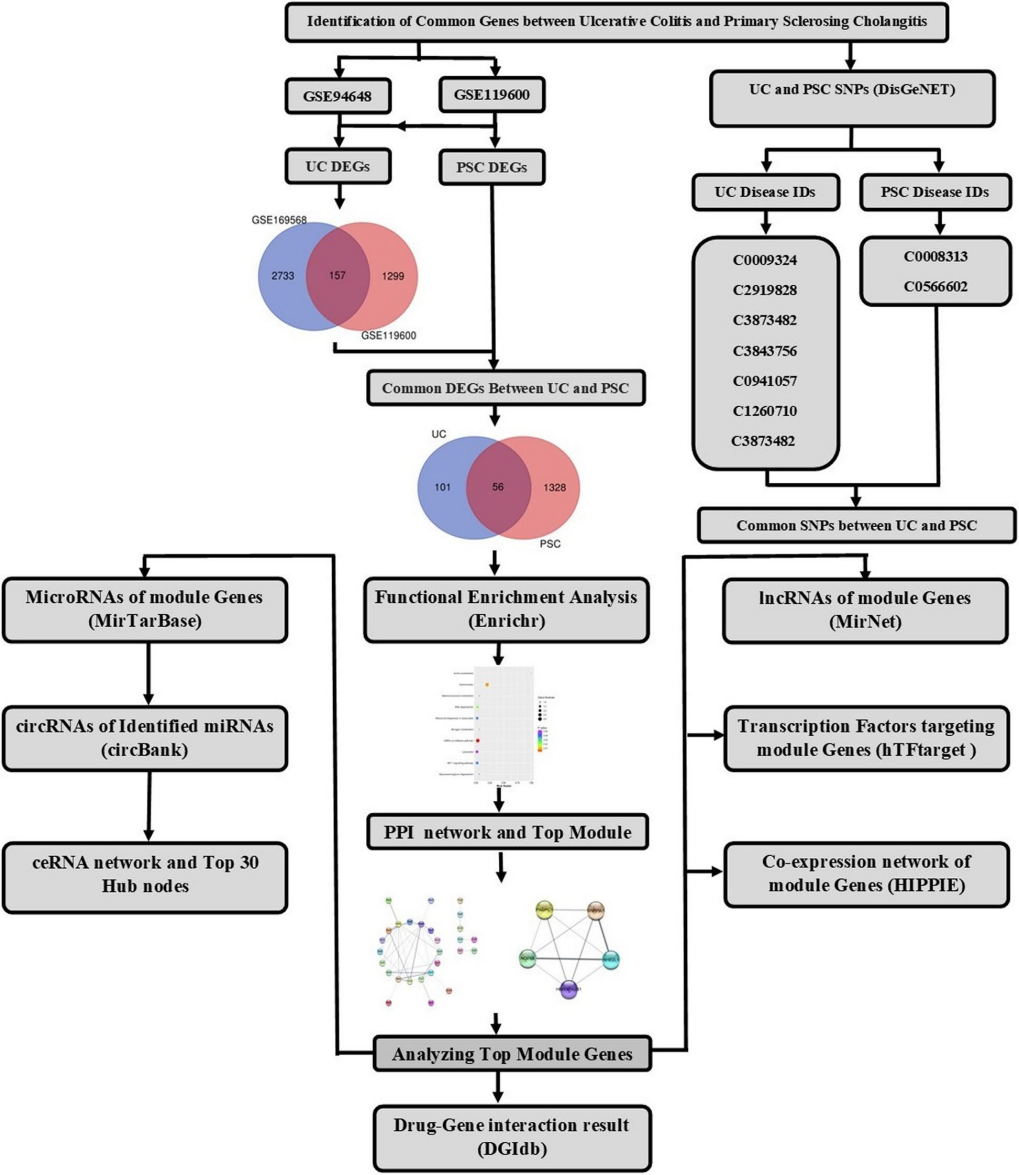
The flowchart of the study. The common differentially expressed genes (DEGs) between UC and PSC were detected using GSE119600 and GSE94648. After enrichment analysis and constructing protein–protein interaction (PPI) network, top module was identified. Then, top module genes were comprehensively and systematically evaluated via multiple bioinformatics databases. Additionally, common SNPs between UC and PSC were identified using DisGeNET database

Common differentially expressed genes (DEGs) between UC and PSC were detected by analyzing two microarray datasets of GEO database. In the following, functional enrichment analysis was performed for these common DEGs to determine whether these set of genes are significantly enriched for a particular biological process, molecular function, or cellular component. Also, we analyzed the protein–protein interaction (PPI) network to identify the top cluster proteins with strongest associations. Subsequently, we evaluated miRNAs, circRNAs, lncRNA, transcription factors, co-expression network, and drug-gene interaction which were previously found to be associated with the top clusters’ genes. Then, ceRNA network was constructed. Additionally, common SNPs between UC and PSC were detected (Fig. 1).

### Identification of common SNPs between UC and PSC (DisGeNET)

To detect common single-nucleotide polymorphisms (SNPs), the UC and PSC were searched in DisGeNET database (https://www.disgenet.org) which is one of the largest available bioinformatics database and provides the genetic data about human disorders [15]. The related DisGeNET IDs for UC and PSC were extracted as shown in Table 1. Moreover, the present study involved the isolation of UC- and PSC-linked genes separately, followed by the identification of common genes associated with both conditions via pairwise analysis using the DisGeNET database. To identify SNPs associated with the risk of UC and PSC, the study employed the disgenet2r package, an analysis tool that allows the identification of relevant SNPs [15, 16]. The common SNPs between UC and PSC were then selected using a DisGeNET cut-off score of > 0.5, while SNPs lacking specific gene names were excluded from further analysis. The study adopted two approaches: first, it determined the overlap between UC- and PSC-associated SNPs and the genes obtained from UC and PSC pairwise analysis, and secondly, it investigated the correlation between UC-associated SNPs and PSC-associated SNPs.

**Table 1:** The list of selected disorders and their DisGeNET codes in the present study

No	Disease name	Disease abbreviation	DisGeNET IDs
1	Primary sclerosing cholangitis	PSC	C0008313 C0566602
2	Ulcerative colitis	UC	C0009324 C2919828 C3873482 C3843756 C0941057 C1260710 C3873482

### Microarray mining for UC and PSC

To assess the common gene expression profile between UC and PSC, we used the data from Gene Expression Omnibus (GEO) (http://www.ncbi.nlm.nih.gov/geo) which fulfilled the following inclusion criteria: (1) containing UC and/or PSC blood samples and (2) minimum sample size of 10 in each group. The exclusion criteria were duplicated research, nonhuman studies, and studies with incomplete data. As a result, only GSE119600 and GSE944648 met our criteria. The GSE119600 (platforms: GPL10558 Illumina HumanHT- 12 V4.0 Expression BeadChip) consisted of 45 PSC, 93 UC, and 47 healthy samples. Further, the GSE94648 (platforms: [HG-U133_Plus_2] Affymetrix Human Genome U133 plus 2.0 Array [CDF: Brainarray HGU133Plus2_Hs_ENTREZG_v18]) consisted of 25 UC and 22 healthy samples (Table 2).

**Table 2:** Characteristic of the studied microarray dataset

No	GSE no	GPL/platform	Samples (*n*)			Sample type	Update (year)	Country	Total
			**UC** **(*n*)**	**PSC** **(*n*)**	**HC** **(*n*)**				
1	GSE119600	GPL10558 Illumina HumanHT- 12 V4.0 expression BeadChip	44	45	47	Blood	2019	Poland	146
2	GSE94648	[HG-U133_Plus_2] Affymetrix Human Genome U133 Plus 2.0 Array [CDF: Brainarray HGU133Plus2_Hs_ENTREZG_v18]	25	-	22	Blood	2017	Spain	47

*UC* ulcerative colitis, *PSC* primary sclerosing cholangitis, *HC* healthy control

We pre-processed the CEL files of Affymetrix microarrays by utilizing the Affy package (http://bioconductor.org/packages/release/bioc/html/affy.html, version 1.74.0) and the Robust Multi-array Average (RMA) method [17] in R software (version 4.4.2, http://www.r-project.org/). The limma package (version 3.52.2) [18] in R software was used to screen DEGs between UC and PSC. The cut-off criteria for the identification of DEGs was a |log2 fold change (FC)| value of ≥ 1 and adjusted *P*-value of < 0.05. Then, Venn diagram was drawn by jvenn tool (http://jvenn.toulouse.inra.fr/app/example.html), and the intersection between the datasets was shown.

### Enrichment analysis

To evaluate DEGs’ potential functions and related pathways, Gene Ontology (GO) enrichment analysis including biological processes (BP), molecular functions (MF), and the Kyoto Encyclopedia of Genes and Genomes (KEGG) was performed, using Enrichr (https://maayanlab.cloud/Enrichr/) database. The threshold was a *P*-value < 0.05.

### PPI network analysis, top cluster, and hub genes identification

STRING database (https://string-db.org) was used to construct PPI network, and results were visualized using Cytoscape software (https://cytoscape.org; version 3.9.1). Further analysis to identify the top cluster among the network was performed by MCODE plug-in based on the following parameters: MCODE scores > 5, degree cutoff = 2, node score cutoff = 0.2, max depth = 100, and k-score = 2. In this study, hub genes were defined as the nodes with a degree > 5 and were extracted.

### MicroRNAs and circRNAs regulating genes and ceRNA network

To determine the miRNAs involved in regulating the expression of the top cluster genes, we utilize the miRTarBase database (https://mirtarbase.cuhk.edu.cn). Next, the circRNAs that target the identified miRNAs were determined. We downloaded the data of the circBank database (http://www.circbank.cn) and used SQL Server to detect certain circRNAs. Then, the top circRNAs were selected based on the total score (TOT score). Subsequently, we constructed ceRNA network between mRNAs, miRNAs, and circRNAs by using Cytoscape software 3.9.1 (https://cytoscape.org) [19]. We also used the CytoHubba package to determine the top ceRNA network, hub nodes, miRNAs, and circRNAs based on the degree method [20].

#### LncRNAs

The lncRNAs associated with regulating the expression of the top module genes were identified using the miRNet database, a miRNA-centric network visual analytics platform (https://www.mirnet.ca/). We extracted the experimentally validated miRNAs from the database miRTarBase, identified the lncRNAs associated with these miRNAs, and conducted a meticulous analysis of their overlap using the Genomics web tool (https://bioinformatics.psb.ugent.be/webtools/Venn/).

### Transcription factor

To detect the transcription factors (TFs) regulating the top cluster genes, we employed hTFtarget database (http://bioinfo.life.hust.edu.cn/hTFtarget#). Then, we identified the overlapped TFs between these genes using the Genomics web tool (https://bioinformatics.psb.ugent.be/webtools/Venn/).

### Co-expression network

The data of the HIPPIE database (http://cbdm.uni-mainz.de/hippie/) was employed to retrieve the co-expression networks of the top cluster genes in UC and PSC. The HIPPIE resource serves as a comprehensive solution for both creating and analyzing protein–protein interaction networks pertinent to a particular research inquiry [21]. The co-expression network was considered in colon and liver tissues. The HIPPIE confidence score was considered 0.01, and analysis was performed at a high confidence level (> 0.73). Also, we used the Cytoscape software 3.9.1 to construct the network.

### DGIDB

In the present study, the interactions between drugs and genes of top cluster were assessed by using Drug Gene Interaction Database (DGIdb) (https://www.dgidb.org/), which provides data on drug-gene interactions from publications, databases, and other web-based sources.

## Results

### Identification of common risk SNPs between UC and PSC

Utilizing the DisGeNET database, we identified 1533 and 452 genes associated with UC and PSC, respectively (Supplementary File 1). Upon further analysis, a total of 291 genes were found to be common between UC and PSC. Additionally, we identified 325 SNPs associated with 243 genes in UC and 311 SNPs associated with 178 genes in PSC. Among them, 132 SNPs were shared between the two diseases, and the genes related to these SNPs were also common between UC and PSC (Supplementary File 1).

### Determination of common DEGs between UC and PSC

**Fig. 2:**
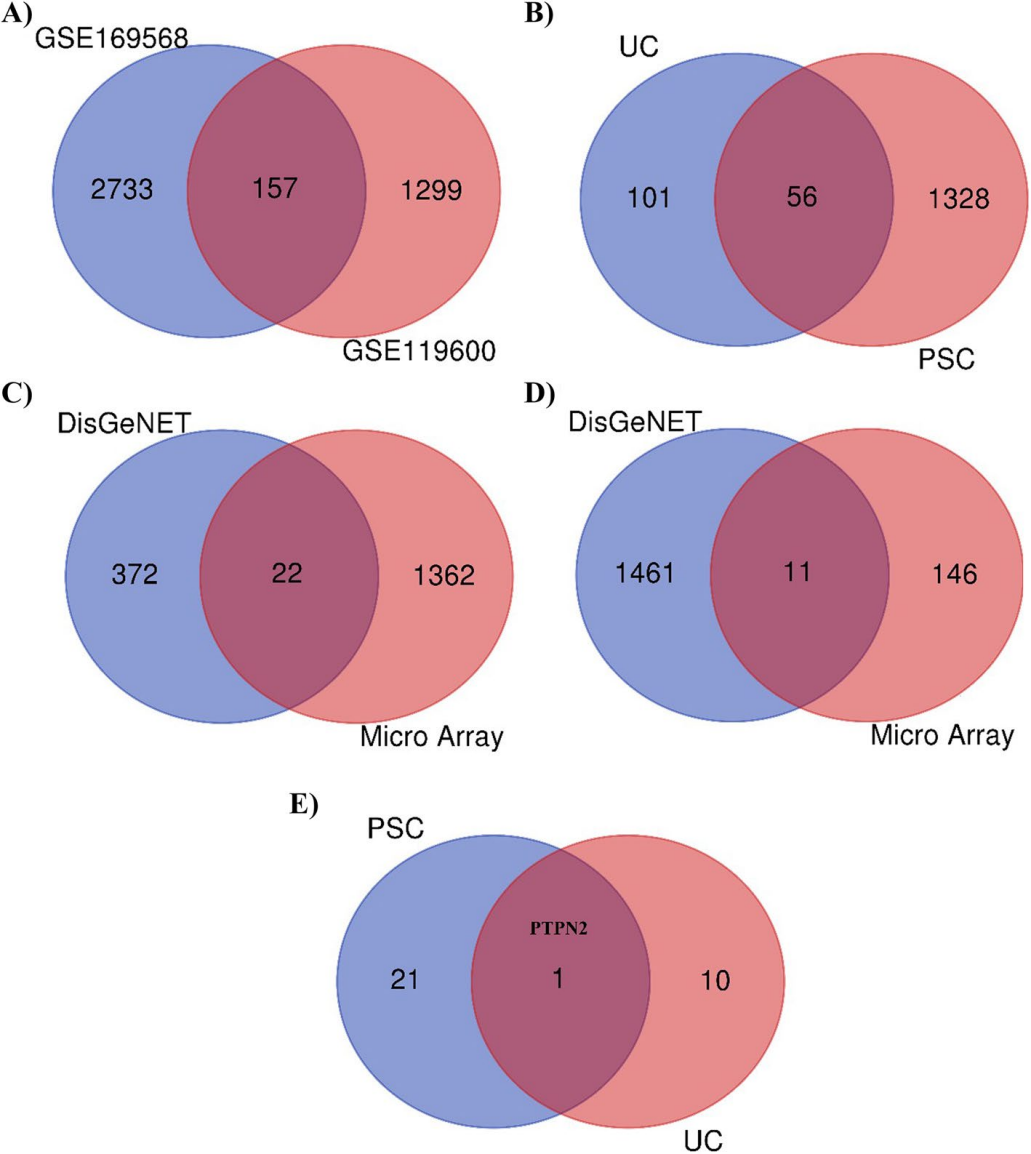
Venn diagram for obtaining shared DEGs. Two GSE datasets were identified for UC and one GSE dataset for PSC. A total of 157 common differentially expressed genes (DEGs) were identified among the two datasets for UC (**A**). Fifty-six common DEGs were detected between UC and PSC (**B**). Moreover, 22 common DEGs were identified for PSC between DisGeNet and microarray datasets (**C**), and 11 common DEGs were further detected for UC between DisGeNet and microarray datasets (**D**). PTPN2 was identified as the overlapped gene between UC and PSC, presenting in both the DisGeNET database and microarray datasets (**E**). UC, ulcerative colitis; PSC, primary sclerosing cholangitis; DEGs, differentially expressed genes

The microarray data were extracted from the GEO database to detect the DEGs in the two selected diseases. Based on the inclusion/exclusion criteria, GSE119600 and GSE169568 were selected, consisting of gene expression profiles in UC, PSC, and normal tissue. According to adjusted *P*-value < 0.05 and log |FC|> 1 as the cut-off criterion, 4189 UC-related DEGs were detected from expression profile datasets of GSE119600 and GSE169568, and 1384 PSC-related DEGs were obtained from expression profile datasets of GSE119600 (Supplementary File 1). There were 157 common DEGs identified among 2 UC GSE datasets (Fig. 2A) which by integrated bioinformatics analysis, 56 common DEGs were significantly differentiated in both UC and PSC patients (Fig. 2B and Supplementary File 1). Additionally, pairwise analysis was conducted to identify the overlapped genes between the DisGeNET database and microarray datasets. The analysis revealed 11 genes that were common to both the DisGeNET database (Fig. 2C) and microarray datasets for UC and 22 genes that were common for PSC (Fig. 2D). Furthermore, PTPN2 was identified as a common gene between UC and PSC presenting in both the DisGeNET database and microarray datasets (Fig. 2E). The PTPN2 gene was downregulated in both UC and PSC. Also, we found two SNPs related to PTPN2 gene, including rs62097857 rs12968719, using DisGeNET database.

### Functional enrichment analysis

**Fig. 3:**
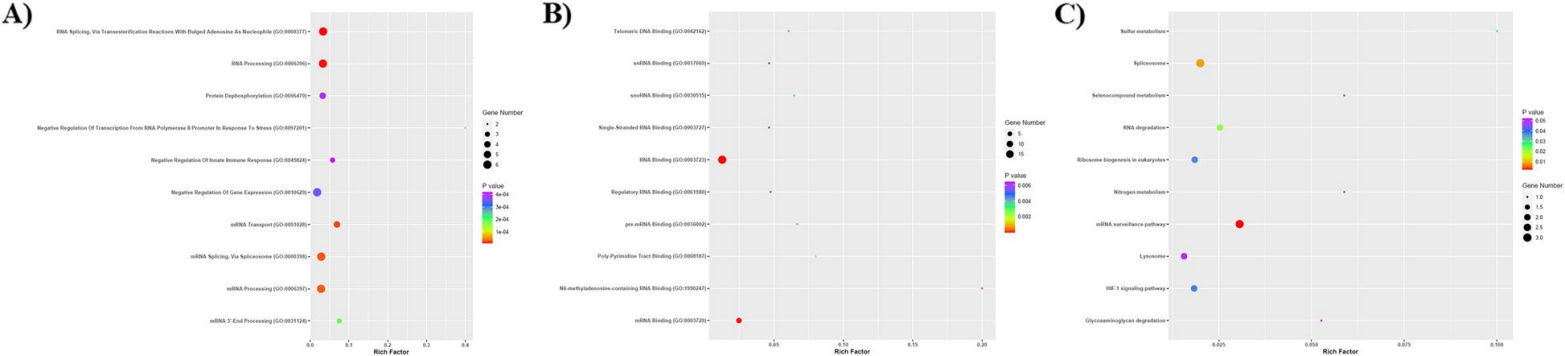
Gene Ontology enrichment analysis. **A** Biological process (BP) in UC and PSC mainly shows the mRNA splicing processes as the dominant process associated with UC-PSC common DEGs. **B** Molecular function (MF) enrichment analysis revealed the dominancy of the RNA bindings in UC and PSC common DEGs. **C** KEGG pathway analysis based on rich factor and *q*-value in UC- and PSC-associated DEGs

The potential biological function of common DEGs was evaluated by GO and KEGG pathway enrichment analyses, and components with *P*-values < 0.05 were selected. A total of 207, 48, and 8 significant BP, MF, and KEGG pathways were identified (Supplementary File 1). Among the related biological processes, mRNA splicing processes (GO: 0000398 and GO: 0000377) and mRNA processing (GO: 0006397) were mostly associated with the common DEGs which YTHDC1, U2 AF2, HNRNPA2B1, SNRPA1, SNU13, and PABPC1 were in these biological processes. Also, common DEGs significantly have roles in regulation of innate immune response which BANF1, CEP63, and PTPN2 had impact on negative regulation of innate immune response (GO:0045824) and CFH, BANF1, and CEP63 had impact on regulation of innate immune response (GO:0045088). Moreover, these genes were mainly enriched in RNA bindings (GO:0003723 and GO: 1,990,247) and mRNA binding (GO:0003729) as molecular functions. The KEGG pathway analysis showed that common DEGs were mostly correlated with spliceosome and mRNA surveillance pathway. The top 10 significant BP and MF and 8 KEGG pathways are shown in Fig. 3A, B, and C.

### PPI and hub genes identification

**Fig. 4:**
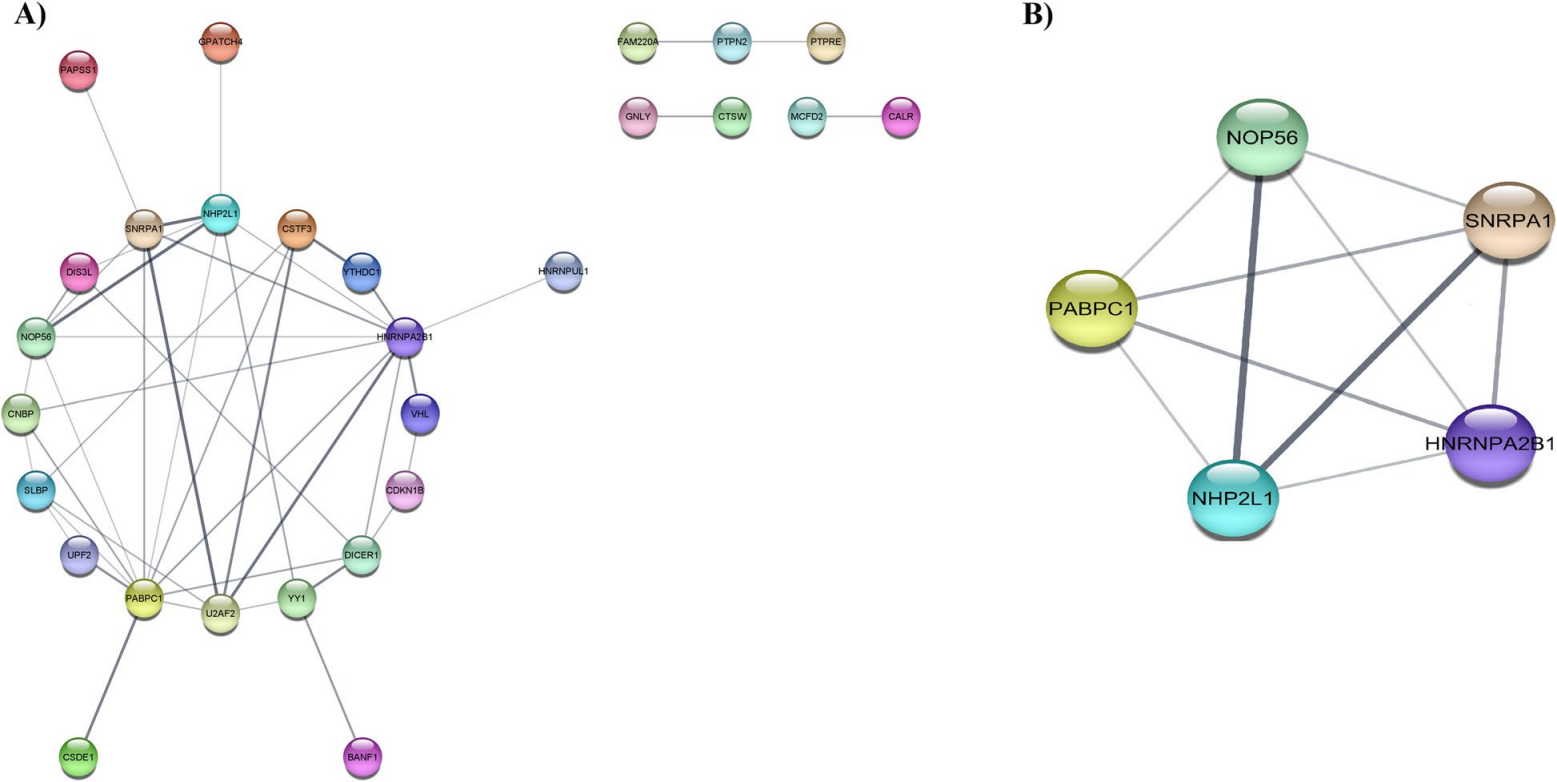
Protein–protein interaction (PPI) network and top module. **A** PPI network of common DEGs between UC and PSC. **B** The most significant cluster (module) of the PPI network. PPI, protein–protein interaction

The PPI network of common DEGs including 56 nodes and 46 edges was constructed using data from STRING (Fig. 4A). Also, top module with 5 nodes and 10 edges was identified (Fig. 4B). Genes with degree > 5 were identified as hub genes which were *PABPC1*, *HNRNPA2B1*, *SNU13*, *NOP56*, and *SNRPA1*.

### ceRNA network construction

**Fig. 5:**
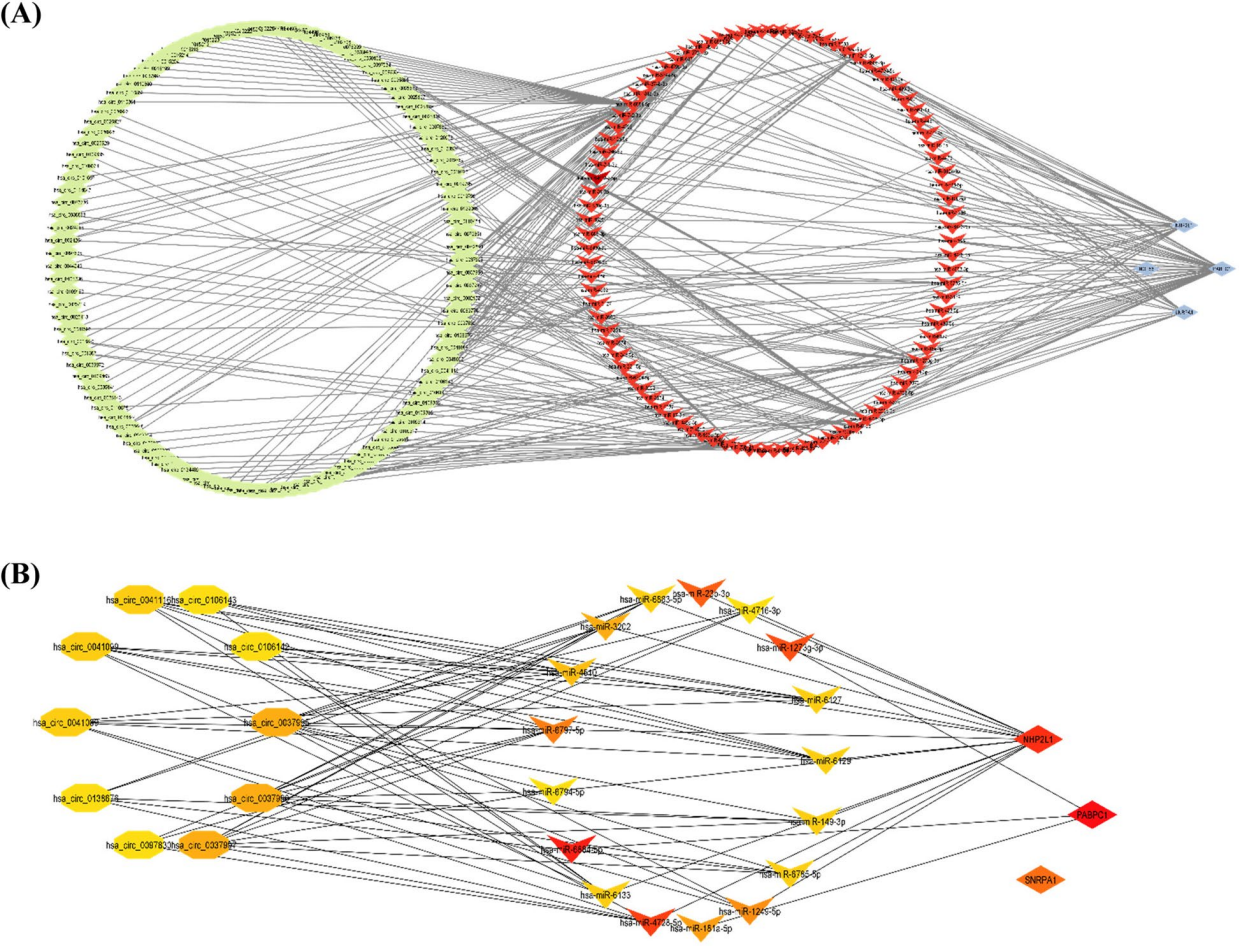
The ceRNA network and hub nodes. **A** The ceRNA network constructed by selected mRNAs, miRNAs, and circRNAs targets. **B** The 30 hub nodes identified by CytoHubba based on degree method; yellow to red refer to the increment of degree value

Using mirTarbase databases, we identified the 94 potential miRNAs involved in regulating the top cluster genes. No miRNAs were identified regulating HNRNPA2B1. Furthermore, 200 circRNAs were detected using circBank database, which are related to the 94 potential miRNAs. The ceRNA network was constructed by the 4 mRNAs, 94 miRNAs, and 200 circRNAs (Fig. 5A). The 30 hub nodes identified by CytoHubba based on degree method include PABPC1 (score: 49), hsa-miR- 6884 - 5p (score: 34), NHP2L1 (score: 27), hsa-miR- 4728 - 5p (score: 25), hsa-miR- 1273 g- 3p (score: 22), hsa-miR- 23b- 3p (score: 18), SNRPA1 (score: 16), hsa-miR- 6797 - 5p (score: 15), hsa-miR- 1249 - 5p (score: 11), hsa-miR- 181a- 5p (score: 9), hsa-miR- 3202, hsa_circ_0037995, hsa_circ_0037996, and hsa_circ_0037997 (score: 8), hsa-miR- 4510 (score: 7), hsa-miR- 6133, hsa-miR- 6127, hsa-miR- 6129, hsa_circ_0041089, hsa_circ_0041099, hsa_circ_0041116, hsa-miR- 6883 - 5p, hsa-miR- 6785 - 5p, and hsa-miR- 149 - 3p (score: 6), hsa-miR- 6794 - 5p, hsa-miR- 4716 - 3p, hsa_circ_0097830, hsa_circ_0138676, hsa_circ_0106143, and hsa_circ_0106142 (score: 5) (Fig. 5B).

Of 94 identified miRNAs, 38 miRNAs were found to be associated with 590 lncRNAs, using the information form miRNet database. We performed an analysis to detect common lncRNAs between those related to each miRNA, which no common lncRNA was found.

### Transcription factors

**Fig. 6:**
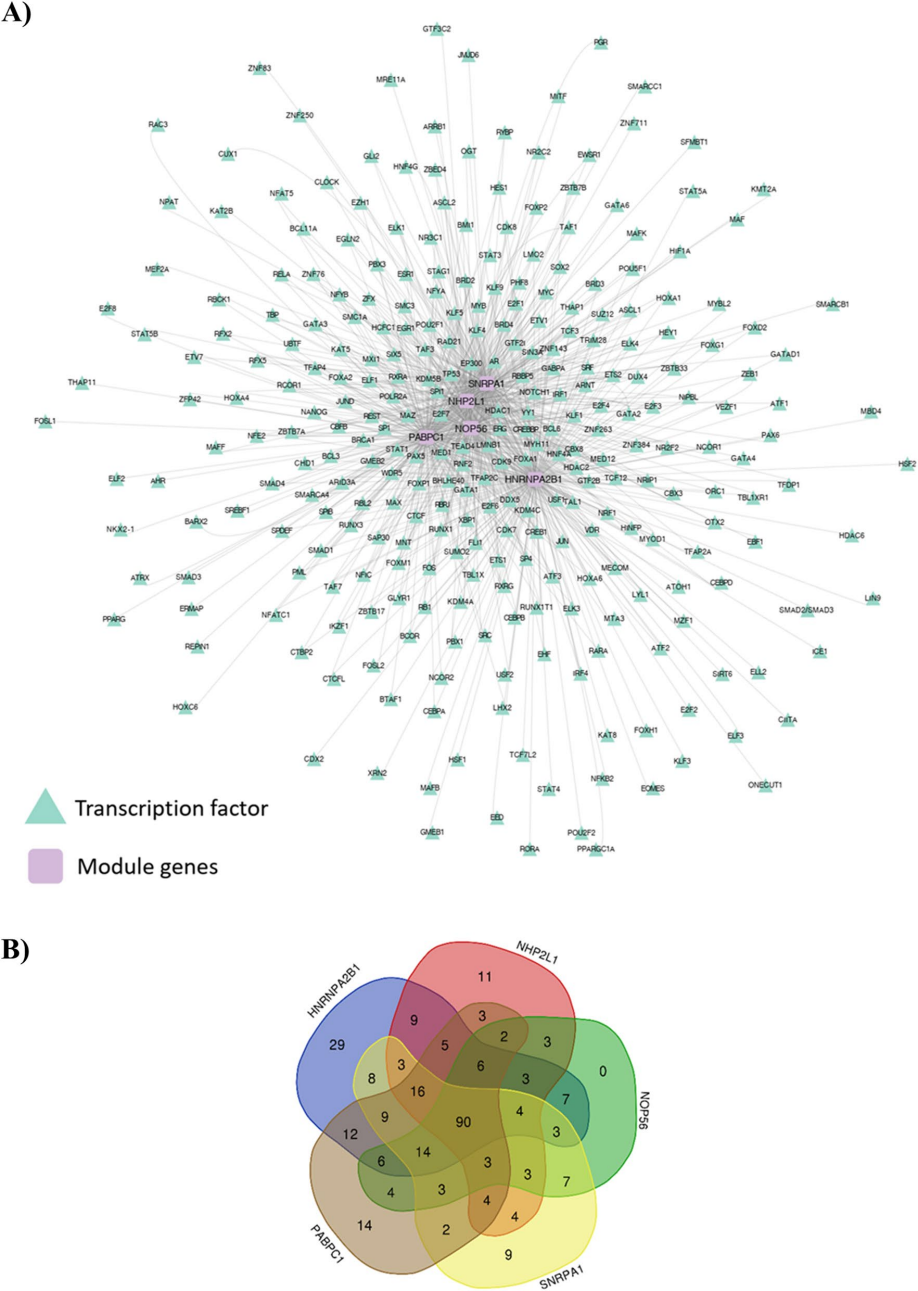
Transcription factors regulating the expression of top module genes. **A** Two-hundred twenty-four TFs for HNRNPA2B1, 169 TFs for NHP2L1, 158 TFs for NOP56, 182 TFs for SNRPA1, and 192 TFs for PABPC1 were identified, which may be involved in regulating these genes. **B** A total of 90 common TFs were found between the 5 genes of the top module

We investigated the possible transcription factors for top module genes using the hTFtarget database. As shown in Fig. 6A, we found 224 TFs for *HNRNPA2B1*, 169 TFs for *NHP2L1*, 158 TFs for *NOP56*, 182 TFs for *SNRPA1*, and 192 TFs for *PABPC1*, which may be involved in regulating the expression of these genes (Supplementary File 1). Further analysis showed that 90 TFs were common between selected genes (Fig. 6B).

### Co-expression network

**Fig. 7:**
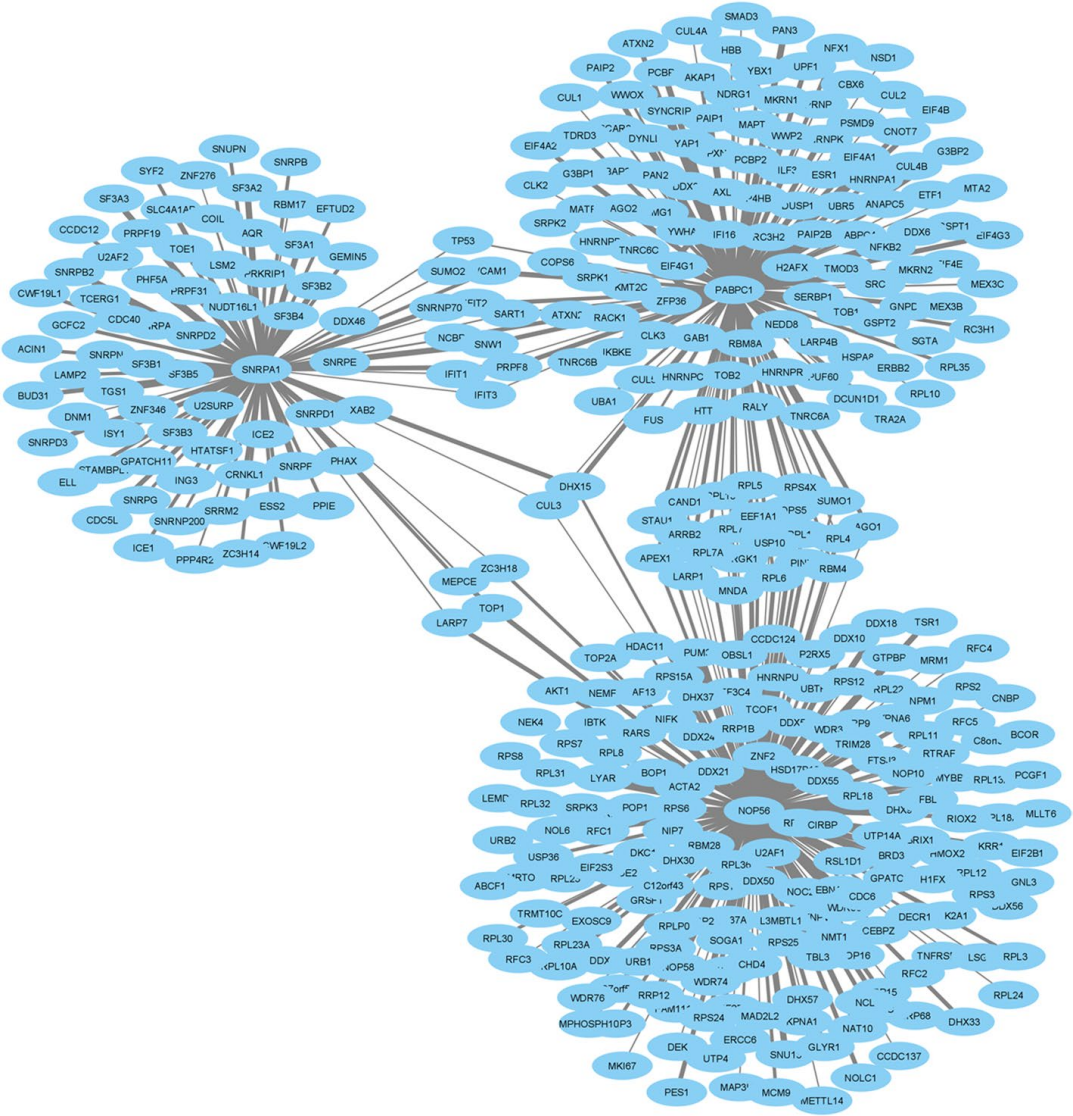
The gene co-expression network of top module genes. The edges with more thickness and dark colors display higher confidence

The co-expression network between the *PABPC1*, *SNRPA1*, *NOP56*, *NHP2L1*, and *HNRNPA2B1* hub genes was identified by using data from HIPPIE database (Fig. 7). This co-expression network revealed that three main subnetworks that are linked together by several genes. Also, two genes, including *CUL3* and *DHX15*, were found to be associated with all three main subnetworks; these associations were stronger for *DHX15* gene. DHX15 is a gene that encodes a putative ATP-dependent RNA helicase, which is reported to play a role in pre-mRNA splicing [22]. DHX15 belongs to the RNA helicase family and is involved in numerous biological processes. Moreover, the CUL3 gene is responsible for coding the cullin- 3 protein. Cullin- 3 is a vital component of an E3 ubiquitin ligase complex that functions through the tagging of excess and defective proteins with ubiquitin molecules, making them recognizable for proteasomes, which lead to their degradation. Additionally, this system regulates the levels of proteins that are involved in critical cellular processes, including the timing of cell division and growth [23, 24].

### Drug-gene interaction result

**Fig. 8:**
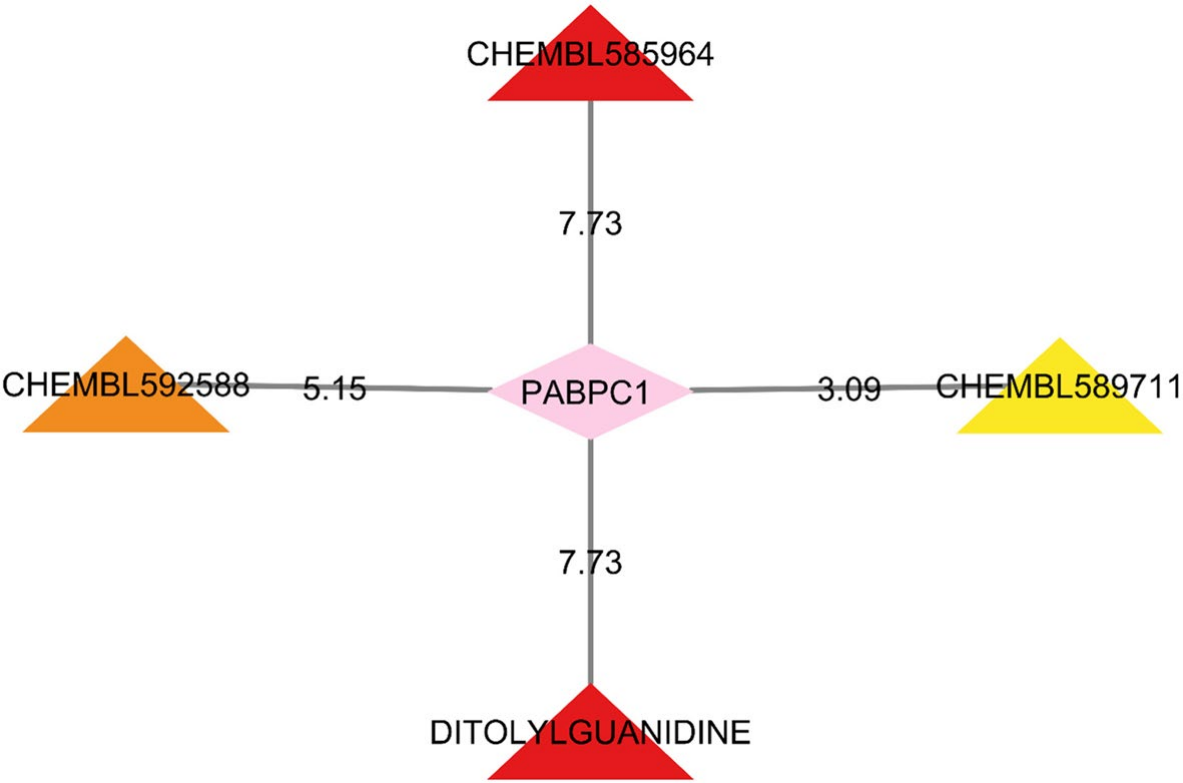
Candidate drugs targeted PABPC1. The nodes are based on degree value; yellow to red refer to the increment of interaction score

Based on the results from DGIdb, we identified a total of four drugs which could potentially have an interaction with *PABPC1* gene, including CHEMBL585964, CHEMBL592588, DITOLYLGUANIDINE, and CHEMBL589711 (Fig. 8). Furthermore, no drugs were identified which have an interaction with SNRPA1, NOP56, and NHP2L1. Moreover, we identified eight potential interacting drugs with PTPN2 gene, including dipicolinic acid, CHEMBL 601167, cloxyquin, CHEMBL 598477, CHEMBL 28721, CHEMBL 599924, CHEMBL 601757, and CHEMBL 578512.

## Discussion and future direction

Primary sclerosing cholangitis (PSC) is one of the most important IBD-associated extraintestinal manifestations (EIMs), which is mostly linked with ulcerative cholangitis (UC), and it is estimated that about 75% of PSC patients also have IBD, primarily UC [25]. However, the exact molecular relationship between IBD and PSC remains unclear. Recent GWAS studies indicated that several genes, which are mostly implicated in immune responses, are associated with these disorders [26]. This strong connection brought us to design a study to explore the molecular relationship of these two conditions using bioinformatics data.

A total of 132 SNPs and their associated genes were found to be shared between UC and PSC which may suggest shared pathogenic mechanisms or biological pathways underlying their co-occurrence. SNPs such as rs10889676, rs11230563, rs11614178, rs12075255, rs12987977, rs1883832, rs183686347, and rs2104286 (related to IL23R, CD6, IFNG-AS1, IL19, IL18R1, CD40, and IL2RA, respectively) were associated with inflammation and immune system in both diseases. Since both ulcerative colitis and primary sclerosing cholangitis implicate the immune system in their pathogenesis, the presence of shared SNPs may indicate a priority role for these SNPs in the regulation of immune and inflammatory response. The imbalance of the immune system initiates a sustained inflammatory response and tissue damage, creating the condition to develop and progress diseases. Ulcerative cholangitis manifests an active immune reaction against gut microbiota which effectively leads to the development and perpetuation of an inflammation in the colon [27]. Abnormal immune-mediated inflammation of the bile ducts in PSC causes fibrosis and ductal obstruction [28]. Because shared SNPs exist between these diseases, it is possible that they regulate immune pathways and inflammation signaling, highlighting the extent to which genetic factors predispose to both diseases.

Gene set enrichment analysis of common 56 identified DEGs showed 207, 48, and 8 significant BP, MF, and KEGG pathways between UC and PSC. Negative regulation of innate immune response and regulation of toll-like receptor 9 signaling pathway were one of the significant BPs. Inflammation in IBD is considered to be the result of activation of the innate immune system inappropriately by the antigens present in the intestinal lumen or by failures in the regulation of its signaling in certain genetic predisposed individuals [29]. Also, TLRs play a main role in the initiation of innate immune responses, and common DEGs identified in these two pathways may have a crucial role in the progression of UC and the advancement of PSC. Likewise, toll-like receptors (TLRs) detect changes in gut microbiota and initiate an immune response, which is crucial in both the development and treatment of IBD [30]. In addition, sulfur metabolism has been identified as a common KEGG pathway between two diseases. Hydrogen sulfide has long been thought of as a possible toxin, with dietary sulfur and the presence of SRB considered relevant contributing factors for H2S production and the progress of UC [31], and sulfur metabolism KEGG pathway might be an important pathway between these conditions. Based on our functional analysis, the common DEGs were mostly involved in mRNA processing, mRNA splicing processes, RNA bindings, and mRNA binding.

Remarkably, the PTPN2 gene was the only gene common between UC and PSC at both the SNP level and the expression level. The protein tyrosine phosphatase nonreceptor 2 (*PTPN2*) gene encodes for the PTPN2 protein, also called T-cell tyrosine phosphatase (TCPTP). PTPN2 protein is a member of the protein tyrosine phosphatases (PTP) family, which regulates the functional activity of their targets through the process of tyrosine phosphorylation, resulting in activating or deactivating cell signaling molecules [32]. This protein is involved in a variety of cellular substrates, including protein tyrosine kinase targets such as the insulin receptor, the epidermal growth factor receptor (EGFR), Src family kinases, as well as multiple types of Janus kinases (JAK), and signal transducer and activator of transcription (STAT) family members [33, 34]. Therefore, PTPN2 plays an important role in cell proliferation, differentiation, growth, mitotic cell cycle, and oncogenic transformation [35]. Additionally, PTPN2 dephosphorylates T-cell receptor kinases, which modifies immune reactions and cellular responses to inflammation, notable in the intestine [36, 37]. Several studies have identified *PTPN2* as a susceptibility gene for IBD, and variations in this gene have been associated with an increased risk of developing chronic inflammatory and autoimmune diseases, such as celiac disease [38], IBD (including both Crohn’s disease and ulcerative colitis) [38, 39], and PSC [40]. These genetic variations may affect the expression or function of *PTPN2*, leading to dysregulated immune responses and increased susceptibility to IBD. For instance, a loss-of-function mutation in the *PTPN2* gene in T cells can lead to enhanced expression and differentiation of naive T-helper cells into Th1 and Th17 subtypes while simultaneously reducing the expression of T regulatory cells. Indeed, patients with *PTPN2* variants exhibited elevated levels of pro-inflammatory cytokines, such as INF-γ, IL- 17, and IL- 22 in both their serum and intestinal mucosal layers [41–43]. Two SNPs of *PTPN2*, namely rs2847297 and SNP rs12968719, have been suggested to be associated with higher risk for PSC [14, 11]. Moreover, a significant correlation between intestinal dysbiosis and exacerbated conditions in individuals with IBD has been unveiled regarding the malfunctioning of *PTPN2* caused by the SNP rs1893217 [44]. Therefore, given the autoimmune inflammatory nature of both UC and PSC, it seems to be reasonable to suggest that the *PTPN2* gene, which significantly influences the inflammation process, is involved in the PSC development in patients with UC. Another study investigated the potential association between the presence of SNP rs1893217 in the *PTPN2* gene and the occurrence of intestinal dysbiosis in patients with UC and PSC. The findings revealed the importance of the gut mucosa-associated microbiome in PSC patients and suggested the role of *PTPN2* rs1893217 as a genetic risk factor for PSC [45]. Our analysis demonstrated two SNPs related to *PTPN2*, including rs62097857 and rs12968719. However, there was no available study regarding the role of these SNPs in UC and/or PSC.

In the next step, we constructed PPI network for common DEGs that five genes were involved in the top module, including *PABPC1*, *SNRPA1*, *NOP56*, *NHP2L1*, and *HNRNPA2B1* which all have an almost common duty in genome expression and RNA modification. The *PABPC1* gene (poly(A) binding protein cytoplasmic 1), located within chromosome region 8q22.2–23, acts as an RNA-binding protein through attaching to the poly(A) tail on mRNA to facilitate the processes of pre-mRNA splicing and maintain the mRNA stability [46]. Importantly, PABPC1 was found to participate in an inhibitory translational complex to suppress the overexpression of pro-inflammatory mediators in activated macrophages [47]. On the other hand, PABPC1 overexpression has also been linked to cancer progression [48, 49] and mediating cell stress responses [50]. The *SNRPA1* gene encodes a protein named small nuclear ribonucleoprotein polypeptide A′, which plays a critical role as a constituent of the U2 small nuclear ribonucleoprotein (snRNP). This complex identifies the branch-point site in the initial stages of pre-mRNA splicing [51, 52]. Additionally, SNRPA1 has been found to inhibit the formation of R-loops, thereby supporting DNA repair processes [51]. Previous research has demonstrated that SNRPA1 functions as a component of the spliceosome and is involved in reprogramming of pluripotent stem cells [53]. In a study, *SNRPA1* was identified as a remarkable factor in inactivating p53, a tumor suppressor gene, in colorectal cancer [54]. The *NOP56* gene plays a pivotal role in processing ribosomal RNA (rRNA) precursors. Reduced expression of NOP56 can impede rRNA biosynthesis, thereby compromising the overall efficiency of rRNA synthesis [55]. Reducing NOP56 expression significantly decreases the growth rate of lung, pancreatic, and colorectal cancer cells harboring KRAS mutations [56].

The *NHP2L1* gene plays crucial roles in telomere maintenance and ribosome biogenesis, both of which are essential for cell viability and proper cellular function [57]. Indeed, protein NHP2L1 was discovered to interact with the U4 RNA, hence is involved in spliceosome assembly, splicing, and gene expression [58]. Dysfunction or mutations in the NHP2L1 gene can lead to telomere shortening, genomic instability, and impaired protein synthesis, potentially contributing to the development of various diseases, including cancer and genetic disorders [59]. The *HNRNPA2B1* gene encodes a critical RNA-binding protein, heterogeneous nuclear ribonucleoprotein A2/B1 (HNRNPA2B1). This protein plays a key role in “reading” RNA methylation modifications and actively participates in downstream processes such as RNA translation and degradation [60]. As a result, *HNRNPA2B1* plays a multifaceted role in various cellular processes, such as telomere function, RNA biology, splicing, correct localization of transcripts, and loading of exosomes [61]. A recent study demonstrated that HNRNPA2B1 exacerbates inflammation by enhancing M1 macrophage polarization, which is mediated by regulating mRNA stability [62]. Another investigation showed the role of the HNRNPA2B1 gene in N6-methyladenosine (m6 A) modification of mRNAs, contributing to IBD pathophysiology [63]. Despite the lack of research in examining these five genes in both UC and PSC, the biological function of these genes, alongside our findings, suggests the potential role of these genes in the UC and PSC relationship.

In further analysis, we identified the 94 potential miRNAs involved in regulating the genes of the top cluster and 200 circRNAs related to the potential miRNAs. We constructed the ceRNA network by the identified mRNAs, miRNAs, and circRNAs and detected 30 RNA molecules with the highest score in this network. Of 94 identified miRNAs, 38 miRNAs were found to be associated with 590 lncRNAs.

A growing body of evidence indicated the essential role of ncRNAs and ceRNA networks in various biological processes, including cell proliferation and growth, apoptosis, immune response, and tissue homeostasis. Therefore, dysregulation of them could contribute to the development of human diseases [64, 65]. Regarding UC, the first miRNA profiling study was conducted in 2008 on colonic tissues of IBD patients. These workers identified 11 miRNAs, which showed differential expression compared to the controls. Among them, 3 and 8 miRNAs were significantly downregulated and upregulated, respectively [66]. Furthermore, Xu S. et al. showed that the circRNA- and miRNA-associated ceRNA network plays a crucial role in various aspects of UC, including the regulation of inflammatory response, immune response, cell proliferation, apoptosis, and progress to tumor formation. This network’s involvement in these processes suggests its significance in the pathogenesis and progression of UC [67]. According to the findings of a study by Ouyang et al., circ_0001187 could exacerbate TNF-α-induced inflammation injury in human normal colorectal mucosa cells through affecting the MYD88 pathway via its interaction with miR- 1236 - 3p [68]. As we identified hsa-miR- 23b- 3p as a hub node in our study, Fasseu et al. found this miRNA as a differentially expressed miRNAs in human CD colonic tissue [69]. Also, hsa-miR- 149 - 3p which is among 30 hub nodes in the present study showed a significant decrease in both active and inactive CD patients compared to the healthy controls in Wu et al.’s study [70]. Povero et al. conducted a study using whole miRNA-sequencing analysis and identified more than 100 different miRNAs in extracellular vesicles derived from patients with PSC. Among these miRNAs, 11 were found to be differentially expressed in extracellular vesicles from PSC patients. The top 5 down-regulated miRNAs were miR- 7113 - 5p, miR- 4715 - 5p, miR- 221 - 5p, miR- 4444, and miR- 150 - 3p. Conversely, the top upregulated miRNAs included miR- 3183, miR- 192 - 5p, miR- 122 - 5p, miR- 4465, miR- 4784, and miR- 4645 - 3p [71]. These differentially expressed miRNAs may provide valuable insights into the molecular mechanisms underlying PSC and potentially serve as diagnostic or therapeutic targets in the future. Nevertheless, none of these miRNAs has been identified as a hub node in our study, which might bring the idea that different miRNAs are involved in connecting PSC to UC. The ongoing research has expanded our knowledge of miRNA and circRNA diversity and their functional significance in various biological processes and diseases. The continuous exploration of miRNAs and circRNAs is crucial for gaining a comprehensive understanding of their regulatory roles and potential therapeutic applications.

We further identified the co-expression network between the hub genes consisting of three main subnetworks linked together by several genes. The *CUL3* and *DHX15* genes were found to be associated with all three main subnetworks; these associations were stronger for the *DHX15* gene. *DHX15* is a gene that encodes a putative ATP-dependent RNA helicase, which is reported to play a role in pre-mRNA splicing [22]. *DHX15* belongs to the RNA helicase family and is involved in numerous biological processes. Moreover, the *CUL3* gene is responsible for coding the cullin- 3 protein. Cullin- 3 is a vital component of an E3 ubiquitin ligase complex that functions through the tagging of excess and defective proteins with ubiquitin molecules, making them recognizable for proteasomes, which leads to their degradation. Additionally, this system regulates the levels of proteins involved in critical cellular processes, including the timing of cell division and growth [23, 24].

According to our findings, several drugs, including CHEMBL585964, CHEMBL592588, ditolylguanidine, and CHEMBL589711, were found to interact with *PABPC1* gene, one of our top cluster genes. Ditolylguanidine is a nonselective sigma receptor agonist which binds to both sigma_1_ and sigma_2_ receptors with equal affinity [72]. In a study, Johannessen et al. focused on exploring how *σ*-receptors modulate the voltage-gated sodium channel (Nav1.5) in the heart. Ditolylguanidine as a sigma_1–2_ receptor ligand reversibly inhibited Nav1.5 channels to multiple degrees in human embryonic kidney 293 (HEK- 293) cells and COS- 7 cells [73]. Despite the novel finding of this study regarding the potential of the ditolylguanidine to be utilized in context of regulating *PABPC1* gene, according to the literature, no study has been conducted in this area. Also, there is no available evidence regarding other detected drugs in our analysis.

Besides the top cluster genes, we extracted possible gene-drug reactions for *PTPN2* as the only common DEG between the DisGeNET and microarray datasets and identified eight potential interacting drugs with this gene. Between these drugs, cloxyquin is a specific activator of the two-pore domain potassium channel TRESK [TWIK-related spinal cord K + channel (also known as K2P18.1)], which is linked to typical migraine occurrence and neuronal excitability control [74]. In fact, TRESK functions to maintain membrane potential in various cells. In this regard, a recent study revealed the pivotal role of physiological TRESK function in the differentiation of T cells toward regulatory subtypes, thus presenting a novel pharmacological target with potential implications for the treatment of autoimmune disorders. Also, TRESK is a crucial mediator in translating the signal from the T-cell receptor during the selection process of thymus-derived regulatory T cells (Treg), ultimately influencing Treg development and function [75]. Hence, the activation of this channel by the compound cloxyquin demonstrates promise as an initial candidate for the development of a novel class of immunomodulatory agents.

At last, our study was constrained by the absence of experimental data to validate these findings and the limited availability of datasets. Although additional verification is needed, our research offers significant insights into potential genes that might play a role in the molecular connection between UC and PSC. This could facilitate the development of diagnostic tools and treatment targets for these conditions.

## Supplementary Information

Supplementary file 1. DisGeNET database.

## Data Availability

All data are available within the manuscript and supplementary files. If additional materials are required, they can be provided upon reasonable request to the corresponding author.

## Abbreviations

BP: Biological process
CD: Crohn’s disease
CeRNA: Competitive endogenous RNA
DEG: Differential expressed gene
DGIdb: Drug Gene Interaction Database
EGFR: Epidermal growth factor receptor
EIMs: Extraintestinal manifestations
FC: Fold change
GEO: Gene Expression Omnibus
GI: Gastrointestinal
GO: Gene Ontology
GWAS: Genome-wide association studies
HNRNPA2B1: Heterogeneous nuclear ribonucleoprotein A2/B1
IBD: Inflammatory bowel disease
JAK: Janus kinases
KEGG: Kyoto Encyclopedia of Genes and Genomes
MF: Molecular function
PPI: Protein-protein interaction
PTP: Protein tyrosine phosphatases
PSC: Primary sclerosing cholangitis
RMA: Robust Multi-array Average
SNPs: Single-nucleotide polymorphisms
STAT: Signal transducer and activator of transcription
TCPTP: T-cell tyrosine phosphatase
TF: Transcription factor
UC: Ulcerative colitis
